# American ginseng suppresses Western diet-promoted tumorigenesis in model of inflammation-associated colon cancer: role of EGFR

**DOI:** 10.1186/1472-6882-11-111

**Published:** 2011-11-09

**Authors:** Urszula Dougherty, Reba Mustafi, Yunwei Wang, Mark W Musch, Chong-Zhi Wang, Vani J Konda, Anirudh Kulkarni, John Hart, Glyn Dawson, Karen E Kim, Chun-Su Yuan, Eugene B Chang, Marc Bissonnette

**Affiliations:** 1 Department of Medicine, University of Chicago, Chicago IL USA; 2 Tang Center for Herbal Medicine Research, and Department of Anesthesia & Critical Care, University of Chicago, Chicago IL USA; 3 Department of Pathology, University of Chicago, Chicago IL USA; 4 Department of Pediatrics, University of Chicago, Chicago, Illinois 60637, USA

## Abstract

**Background:**

Western diets increase colon cancer risk. Epidemiological evidence and experimental studies suggest that ginseng can inhibit colon cancer development. In this study we asked if ginseng could inhibit Western diet (20% fat) promoted colonic tumorigenesis and if compound K, a microbial metabolite of ginseng could suppress colon cancer xenograft growth.

**Methods:**

Mice were initiated with azoxymethane (AOM) and, two weeks later fed a Western diet (WD, 20% fat) alone, or WD supplemented with 250-ppm ginseng. After 1 wk, mice received 2.5% dextran sulfate sodium (DSS) for 5 days and were sacrificed 12 wks after AOM. Tumors were harvested and cell proliferation measured by Ki67 staining and apoptosis by TUNEL assay. Levels of EGF-related signaling molecules and apoptosis regulators were determined by Western blotting. Anti-tumor effects of intraperitoneal compound K were examined using a tumor xenograft model and compound K absorption measured following oral ginseng gavage by UPLC-mass spectrometry. Effects of dietary ginseng on microbial diversity were measured by analysis of bacterial 16S rRNA.

**Results:**

Ginseng significantly inhibited colonic inflammation and tumorigenesis and concomitantly reduced proliferation and increased apoptosis. The EGFR cascade was up-regulated in colonic tumors and ginseng significantly reduced EGFR and ErbB2 activation and Cox-2 expression. Dietary ginseng altered colonic microbial diversity, and bacterial suppression with metronidazole reduced serum compound K following ginseng gavage. Furthermore, compound K significantly inhibited tumor xenograft growth.

**Conclusions:**

Ginseng inhibited colonic inflammation and tumorigenesis promoted by Western diet. We speculate that the ginseng metabolite compound K contributes to the chemopreventive effects of this agent in colonic tumorigenesis.

## Background

Colon cancer arises from activating mutations in oncogenes and inactivating mutations in tumor suppressor genes. While hereditary forms of this disease arise from germline mutations such as loss of function mutations in the *adenomatous polyposis coli *(*apc*) gene, most colon cancers are sporadic and involve somatic mutations in *apc *or other genes. Environmental especially dietary factors are believed to contribute substantially to the risk of colon cancer development [1,2]. In this regard Western diets that are rich in Western style fats have been shown to promote colon cancer in experimental models [2,3]. We recently showed that epidermal growth factor receptors (EGFR) were required for tumor promotion by Western diet [4,5]. In the presence of a Western diet, EGFR signals increased proto-oncogene MYC and pro-inflammatory cyclooxygenase-2 (Cox-2). Since therapies for advanced cancers have limited efficacy, increasing attention has focused on chemopreventive approaches. Efforts to inhibit up-regulated EGFR signals that occur with Western diets might provide such a strategy to prevent these cancers.

Complementary and alternative medicines are widely used for a variety of health purposes. Many of these agents are well tolerated and some have served as lead compounds for developing more effective anti-cancer agents. Several retrospective case-control studies in Korea have supported chemopreventive effects of ginseng that appears to exert a broad spectrum of anti-tumor activities in humans [6-9]. Ginseng is a deciduous perennial plant belonging to the Araliaceae Ivy family that has been used for centuries in China and Korea as an anti-inflammatory agent [10]. Ginseng extracts contain ginsenosides as the major biologically active constituents, which are glycosides with a dammarane skeleton [11]. Many *in vitro *studies have demonstrated anti-tumor effects of ginseng alone or in combination with anti-cancer agents [12-15]. Antioxidant, anti-proliferative and anti-inflammatory effects of ginseng have been identified that may mediate the anti-tumor effects of this herb [16,17]. In addition, ginseng metabolites have been shown to inhibit EGFR-induced epithelial cell growth [18]. Moreover, a recent study in a model of colitis-associated colon cancer showed that ginseng reduced levels of phospho-active EGFR and phospho-active ErbB2 as well as ERK, a down stream effector of EGFR, indicating ginseng could suppress EGFR signals in colonic tumorigenesis [19]. Given the requirement for EGFR in tumor promotion by Western diet, in the current study we investigated the ability of ginseng extract to inhibit colonic tumorigenesis under conditions of Western dietary stress. Tumors were induced with azoxymethane (AOM) followed by dextran sulfate sodium (DSS). Azoxymethane is a pro-carcinogen that is metabolized in the liver and further metabolized in the colon to an active alkylating agent, presumably a methyl carbonium ion [20]. This methyl donor leads to guanine methylations and eventually G to A transitions [21]. Proto-oncogenes targeted by AOM include activating mutations in β-catenin and K-Ras [21]. DSS is a polysulfated polymer that arrests colonic crypt cell re-generation leading to acute mucosal ulceration and clinical colitis that enhances tumorigenesis [22].

A number of studies have identified several bacterial metabolites of ginseng with biological activities [23-26]. These include 20-O-β-(D-glucopyranosyl)-20(S)-protopanaxadiol or compound K that induces apoptosis in colon cancer cells [27-29]. Since the microbiome is essential for compound K generation, we examined the effects of dietary ginseng on microbial diversity and effects of broad-spectrum antibiotics on compound K bioavailability. Several ginsenosides have been shown to inhibit cancer growth, including colon cancer cells in tumor xenograft models [15,30-34]. To directly test compound K anti-tumor activity in colon cancer, we also examined the effects of this microbial metabolite of ginseng on colon cancer cell growth in a tumor xenograft model.

Taken together, in this study we demonstrate for the first time that ginseng can inhibit inflammation-associated colonic tumorigenesis in mice fed a tumor-promoting Western diet. Furthermore, compound K, a bacterial metabolite of ginsenoside Rb1, directly inhibits colon cancer cell growth *in vivo*.

## Methods

### Materials

HCT116 cells were obtained from ATCC (Manassas, VA). Male A/J and immuno-deficient *nu/nu *mice were obtained from Jackson Laboratories (Bar Harbor, ME). Azoxymethane was obtained from Midwest Research (Kansas City, MO), the NCI Chemical Carcinogen Reference Standard Repository. The sodium salt of dextran sulfate (MW 36,000-50,000) was obtained from MP Biomedicals (Solon, OH). Ginseng extract was obtained from Wisconsin Ginseng Board (Wausau, WI). Compound K (20-O-β-D-glucopyranosyl-20(S)-protopanaxadiol) was purchased from ChromaDex (Irvine, CA). The molecular weight of compound K is 6,222.87. Harlan Teklad (Madison, WI) prepared AIN-76A and Western (20% fat) and control chow diets. Details of the diet were as described [35]. RC-DC protein assay was obtained from Bio Rad (Hercules, CA). Rabbit polyclonal anti-Cox-2 antibodies (#160106) were purchased from Cayman Chemicals (Ann Arbor, MI). Polyclonal antibodies to pAKT (#9271) were obtained from Cell Signaling (Danvers, MA). Antibodies to pEGFR (SC-16802), pErbB2 (SC-12352R), pERK (SC-7383), Bax (SC-493), Bcl2 (SC-7382), c-Jun (SC-1694) and cyclin D1 (SC-718) were obtained from Santa Cruz Biotechnology (Santa Cruz, CA). Antibodies to phospho-active EGFR (SC-16802) recognize human EGFR phosphorylated tyrosine 1092 and antibodies to phospho-active ErbB2 (SC-12352-R) recognize human ErbB2 phosphorylated tyrosine 1248. Monoclonal antibodies to β-actin were purchased from Sigma-Aldrich (St. Louis, MO). Antibodies to p27Kip1 and p21Waf1 were purchased from BD Transduction Laboratories (San Jose, CA). PCNA antibodies were obtained from Biomeda Corp. (Foster City, CA). TUNEL assay kit was purchased from Millipore (Billerica, MA).

### Methods

#### Ginseng extraction and chemical analysis

American ginseng root powder was obtained from Wisconsin Ginseng Board (Wausau, Wisconsin). Five hundred grams of ginseng was extracted in 10 vol 70% ethanol under reflux for 2 h in a water bath kept at 90-95°C. The filtrate was collected and the extraction procedure repeated on the residue. The residue was discarded after the second extraction. The filtrates were combined and passed through filter paper. Filtrate was evaporated under vacuum. The extract was dissolved in 500 ml water and extracted 4 times (125 ml × 4) in water-saturated n-butanol. The n-butanol phase was evaporated under vacuum, and lyophilized. All solvents used for extraction were US pharmacopoeia (USP) purity. The ratio of starting material (dried American ginseng) to final ginseng extract was approximately 10:1. The ginsenosides in the extract were characterized by UF-HPLC as described [14,36]. The ginsenoside concentrations (mg/gram extract) were Rg1, 14.0; Re, 197.9; Rb1, 341.8; Rc, 34.2; Rb2, 4.6; Rb3, 6.8; Rd, 65.0; Rg3, 0.6 as reported [37].

#### Tumor Induction

**Figure 1:**
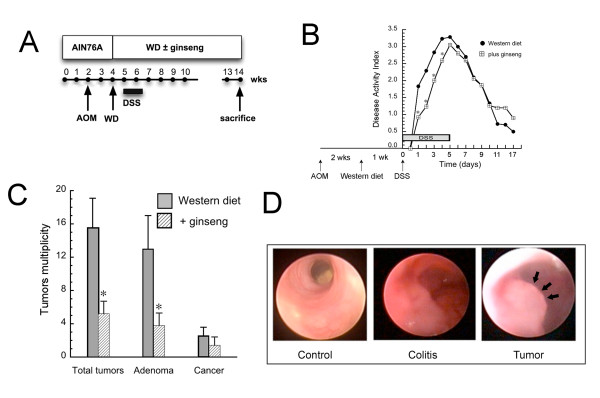
**Ginseng inhibits colitis and colonic tumorigenesis in mice on Western diet**. **A**. Protocol for tumor induction. Mice received AOM at wk 2. WD was initiated at wk 4 and DSS was given beginning in wk 5. Mice were sacrificed in wk 14. **B**. Time course of DSS colitis. Animals were treated as shown in Figure 1A. DSS induced colitis was scored as disease activity index (DAI) based on weight loss, diarrhea and bleeding as described [38]. Values were expressed as mean ± SD (n = 7 AOM/DSS, n = 5 AOM/DSS + ginseng; *p < 0.05, compared to Western diet alone). Ginseng significantly delayed onset of colitis and appeared to limit peak inflammation. **C**. Tumor multiplicity. Mice were treated as described in Figure. 1A and sacrificed 12 wks after AOM. Tumor histology was determined by standard criteria. Tumor multiplicity was calculated using the Wilcoxon-Mann-Whitney test (n = 7 AOM/DSS, n = 5 AOM/DSS + ginseng; *p < 0.05 compared to AOM/DSS alone). Note that ginseng significantly inhibited total tumor multiplicity and the number of adenomas/mouse. **D**. Colonoscopy detects colitis and tumors. Shown are representative colonoscopic views of control mice (*left panel*), mice with colitis 5 days after DSS (*middle panel*) and mice with tumors 12 wks after AOM (*right panel*). Note the increased mucosal erythema in the colitis-bearing mouse and intraluminal mass in the tumor-bearing mouse (black arrows).

Use of animals for these studies was approved under the guidelines of the Institutional Animal Care and Use Committee (IACUC) at University of Chicago, which complies with the guidelines outlined by the National Institutes of Health. A/J male mice weighing 20-22 grams were acclimated for 2 wks on AIN-76A chow. We followed a modified protocol to induce colitis-associated colon cancer [22]. Mice received a single dose of azoxymethane (AOM) 10 mg/kg body weight or saline (AOM vehicle). Two wks later mice were randomized to receive a Western diet (20% fat) or Western diet supplemented with 250-ppm ethanol/butanol extracted American ginseng. Western diet was 20% fat and included beef tallow (35 gm/kg), lard (30 gm/kg) and corn oil (80 gm/kg). Detailed composition of WD was reported [35]. We calculated that the ginseng dose was approximately 0.875 mg ginseng extract/mouse/day. One wk after starting Western diet, mice received 2.5% DSS in the drinking water × 5 days. DSS-induced clinical colitis was assessed as described [38]. It should be noted that there were no endoscopically visible tumors for several weeks after DSS. Mice were sacrificed 12 wks after AOM treatment and tumors and colonic mucosa harvested. The protocol is summarized in Figure 1A. A/J mice are very sensitive to AOM [39], and thus only one dose of AOM followed by one cycle of DSS was sufficient to induce tumors in these mice. Fifteen min prior to sacrifice mice were treated with peroxovanadate as described to lock in phospho-EGFR signals [40,41]. Tumors were divided and one aliquot was fixed in 10% buffered formalin for histology. Colonic mucosa and another aliquot of tumor were flash frozen for Western blotting.

#### Tumor histology

Tumor histological features were assessed by a GI pathologist (JH). Adenomas were defined as neoplastic lesions characterized by abnormal glandular architecture with branching or elongated crypts. Cytologically, these lesions exhibited hyperchromatic, elongated and pseudostratified nuclei with mildly increased nuclear to cytoplasmic ratios. Adenomas with carcinoma *in situ *exhibited marked glandular complexity, including areas with a cribriform configuration and marked cytological atypia, characterized by large irregular nuclei and loss of nuclear polarity.

#### Mouse colonoscopy

Colonoscopy was performed using Karl Storz-Endoskope to monitor colitis and tumor development as described [42]. The colonoscope was advanced 4 cm proximal to the anus. If needed, water was used to irrigate the colon. In some cases, the colonoscope was advanced to the cecum if there was no resistance. Careful visual inspection with white light was performed upon withdrawal. During endoscope withdrawal, lesions were described and endoscopic pictures captured. External 5 mm marking on the colonoscope were used to estimate lesion location within the colon.

#### Western Blotting

Proteins were extracted in SDS-containing Laemmli buffer, quantified by RC-DC protein assay and subjected to Western blotting as described (4). Briefly, proteins were separated by SDS-PAGE on 4-20% resolving polyacrylamide gradient gel and electroblotted to PVDF membrane. Prestained molecular markers were included in each gel. Blots were incubated overnight at 4°C with specific primary antibodies followed by 1 hr incubation with appropriate peroxidase-coupled secondary antibodies that were detected by enhanced chemiluminescence using X OMAT film. Xerograms were digitized using an Epson scanner (San Jose, CA) and band intensity quantified using UN-SCAN-IT gel software (V 5.3, Silk Scientific, Orem UT). Protein levels in tumors were normalized to β-actin levels and expressed as fold of control colonic mucosa (means ± SD). Protein lysates from tumors and control colonic mucosa with equal protein abundance as assessed by RC-DC assays showed comparable β-actin levels by Western blotting. Tumors of comparable stage (adenomas) were used for Western blotting comparisons.

#### Immunostaining

Five-micron sections were mounted on Vectabond-coated Superfrost Plus slides. Sections were heated to 60°C for 1 hr, deparaffinized by 3 washes × 5 min in xylene, hydrated in a graded series of ethanol washes and rinsed in distilled water. Epitope retrieval for Ki-67 was achieved by pressure cooker for 15 min in Tris-EDTA buffer, pH 9 followed by 3 washes × 2 min in Tris-buffered saline with 0.1% Tween-20 (TBST). Endogenous peroxidase activity was quenched with methanol/H2O2 solution (0.5%). Sections were washed 3 times in TBST × 2 min and blocked in Protein Block for 20 min. Sections were incubated with 1:300 dilution of anti-Ki67 antibodies for 1 hr at room temperature. After 3 TBST washes, slides were incubated at room temperature with 1:200 dilution of biotinylated secondary antibodies for 30 min. Antigen-antibody complexes were detected using an HRP labeled DAKO EnVision™+ System and 3,3'-diaminobenzidine as substrate. For negative controls, sections were incubated with isotype matched non-immune antibodies. After washing in distilled water, slides were stained with Gill's III hematoxylin, rinsed with water, dehydrated in ethanol and cleared with xylene. For TUNEL assay, epitopes were retrieved by treatment with protease1 digestion for 10 min at room temperature. After blocking endogenous peroxidases with hydrogen peroxide, tissues were incubated in equilibration buffer and treated with terminal deoxynucleotidyl transferase (TdT) enzyme to detect TUNEL-positive nuclei as suggested by the manufacturer (Roche Scientific, Indianapolis IN). Tissues were then incubated with peroxidase-conjugated anti-digoxigenin antibodies and color developed with diaminobenzidine (DAB). After counterstaining with methyl green, sections were protected with cover slip secured with mounting medium. Tumors of comparable histology (adenomas) were used for all immunostaining comparisons.

#### Immunostaining Quantitation

Ki67 nuclear staining and TUNEL positive cells were quantified by the automated Aperio Scanning imaging system (Vista CA). Proliferation was expressed as % nuclei positive for Ki67. Color-specific thresholds were used to determine brown (Ki67 positive) and blue (Ki67 negative) nuclei within the outlined regions of interest to calculate the fraction of positively stained nuclei. Cell death was scored as % nuclei positive for TUNEL. At least 5 fields per tumor and 3 tumors per group (~50,000 cells/condition) were scanned for quantitation.

#### Tumor Xenografts

For tumor xenograft studies, HCT116 cells obtained from ATCC were cultured as described previously [5]. Cells (5 × 10^6) were implanted subcutaneously into the flanks of athymic immunodeficient nude mice (nu/nu mice). Tumors were allowed to grow for one week prior to treatment with compound K or vehicle (DMSO). Mice were treated daily with compound K (30 mg/kg body wt) or DMSO. Tumor dimensions were measured serially with a vernier calipers and tumor volume calculated as (width)^2 ^× length/2. Tumor size was estimated from volumes assuming a density of 1 gram/ml. Mice were sacrificed and tumors harvested 4 wks after implantation.

#### Analysis of microbial 16S rRNA

Mice were randomized to receive Western diet or Western diet supplemented with 250 ppm ginseng. Two weeks after starting on the diet, fresh stool was collected and bacterial DNA extracted. Clone library preparation and sequencing analyses of bacterial genes encoding 16S rRNA were performed as described [43]. 16S rRNA gene sequences were amplified from DNA samples using primers 8F (5'-AGAGTTTGATCCTGGCT-CAG-3') and 1492R (5'-GGTTACCTTGTTACGACTT-3') for the conserved domain of bacterial 16S rRNA. PCR reactions were performed for 30 cycles using Takara high-fidelity Ex Taq (Takara Mirus Bio, Madison, WI, USA) with an annealing temperature of 58°C. PCR products were purified by QIAquick gel extraction kit (Qiagen, Valencia, CA) and cloned into pCR-2.1-TOPO^® ^vectors (Invitrogen, Carlsbad, CA) using the TOPO-TA Cloning Kit according to the manufacturer's instructions. From each library, 100 colonies were picked randomly and processed for DNA sequencing using 8F as the sequencing primer.

#### Sequence alignment and phylogenetic analysis

The 16S rRNA gene sequences were analyzed as described previously [43]. Briefly, raw sequence data were processed by base-calling, quality-trimming and alignment, using the RDP pipeline server at the Ribosomal Database Project II (RDP-II) website http://rdp.cme.msu.edu/pipeline. Potential chimeric sequences were checked and excluded as appropriate using the SimRank 2.7 package available through the RDP. The RDP-II classifier analysis tool and NCBI BLAST tool were used to assign 16S rRNA sequences to the taxonomical hierarchy at phylum level. For principal coordinate analysis (PCA), all 16S rRNA gene sequences were imported using the ARB software package and aligned into a phylogenetic tree, which was used to perform clustering analysis without abundance weighting using online UniFrac. All sequences will be deposited in the GenBank nucleotide sequence databases post-publication.

#### Measurements of Rb1 and compound K in mouse sera

Mice were given unsupplemented drinking water or drinking water supplemented with metronidazole (600 μg/ml) for 5 days. Mice were then gavaged with 500 mg/kg ginseng extract. At indicated times mice were sacrificed and blood obtained for plasma measurements of ginsenoside Rb1 and compound K by UPLC/MS-TOF analysis. Mouse plasma was mixed with 20 μL of internal standard (digoxin, 10 μg/ml). The plasma sample was diluted with 1 ml of saline and purified by solid-phase extraction (SPE) with a Sep-Pak C8 Vac 3cc 500 mg cartridge (Waters, Milford, MA). The purified eluate, containing ginsenosides and metabolites, was evaporated to dryness at 40°C under nitrogen and dissolved in 200 μL methanol. Chromatographic separation was performed on an Agilent 1200 series (Agilent, Germany) liquid chromatographic system at 25°C using an Agilent ZorBax Extend-C18 UPLC column (50 × 2.1 mm, 1.8 μm). The mobile phase consisted of 0.1% formic acid in water (A) and acetonitrile (B). Gradient elution started with 82% solvent A and 18% solvent B, changed to 21% B for 6 min, then changed to 26% B for 1 min and held for 2 min; changed to 27% B for 4 min and held for 1 min; changed to 30% B for 3 min; changed to 36% B for 5 min; changed to 50% B for 3 min and held for 2 min; changed to 33% B for 5 min. The flow rate was kept at 0.4 ml/min, and the sample volume injected was set at 2 μL. The TOF/MS analysis was performed in full-scan mode with an electrospray ionization (ESI) source and the mass range was set at mass to charge ratio (m/z) 100-1500 in negative mode. The acquisition and analysis of data were controlled by Agilent LC-MS TOF software, version A.01.00 and Applied Biosystems/MDS-SCIEX Analyst QS software, respectively. A calibration curve was used to calculate the compound concentration in samples. The curve plots the concentration of the standard (x-axis) against the area ratio of Rb1 or compound K/internal standard (y-axis). The measured sample area ratio then allows estimation of sample concentration from the calibration curve.

#### Statistical Methods

Continuous data were expressed as means ± SD, and compared using Student's t-test. Tumor multiplicity was defined as the average number of tumors in a given group. Tumor multiplicity was compared using negative binomial regression and significance calculated using the Wilcoxon-Mann-Whitney test [44]. All statistical analyses were performed using Stata v. 10, and p-values < 0.05 were considered statistically significant.

## Results

### Ginseng inhibits AOM/DSS-induced colitis and tumorigenesis under Western diet

The study protocol is summarized in Figure 1A. As expected DSS induced clinical colitis as assessed by disease activity index that was scored as described [38]. As shown in Figure 1B, ginseng significantly delayed the onset of DSS-induced clinical colitis and appeared to reduce maximal inflammation. At the time of sacrifice there was minimal histological inflammation present. Ginseng significantly reduced tumor multiplicity from 15.6 ± 3.1 in the Western diet group to 5.1 ± 2.3 (p < 0.05) in the group receiving Western diet and ginseng (Figure 1C). There were also significantly fewer adenomas in the ginseng group (p < 0.05), and a trend towards fewer cancers (p = 0.08) as shown in Figure 1C. During the study we examined mice by colonoscopy to monitor inflammation and tumor development. Representative colonoscopic views of control mice, mice with colitis and mice with colonic tumors are shown in Figure 1D.

### Ginseng inhibits proliferation and increases apoptosis in AOM/DSS tumors

**Figure 2:**
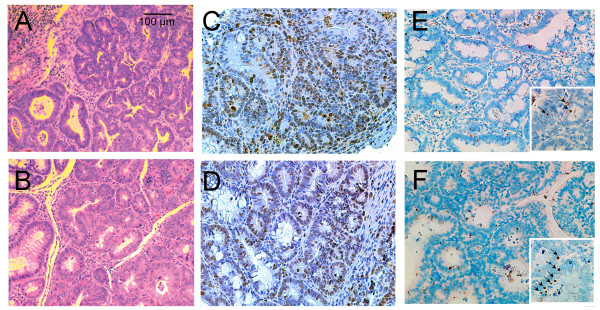
**Ginseng reduces proliferation and increases apoptosis in AOM/DSS-induced tumors from mice fed a Western diet**. **A**. H&E stained tumor from mouse on Western diet alone; (20×); **B**. H&E stained tumor from mouse on Western diet supplemented with 250 ppm Wisconsin ginseng extract (20×); **C**. Ki67 in tumor from WD fed mouse, (20 x); **D**. Ki67 in tumor from mouse on WD plus ginseng (20×). Note the increased brown staining (Ki67 positive nuclei) in C compared to D; **E**. TUNEL staining in tumor from WD fed mouse (20×); **F**. TUNEL staining in tumor from mouse on WD plus ginseng (20×). *Inset *40× magnification. Note the increased apoptotic bodies indicated by black arrows in F compared to E.

To elucidate the effects of ginseng on proliferation and apoptotic cell death we examined tumors for Ki67 and TUNEL staining. As shown in Figure 2, ginseng reduced proliferation and increased apoptotic cell death. Quantitation of proliferation and cell death in colonic tumors are summarized in Table 1. Ginseng caused nearly a 50% reduction in proliferation and nearly a 50% increase in apoptosis.

**Table 1:** Ginseng inhibits proliferation and increases cell death in colonic tumors

Group	Ki67 staining (% positive nuclei)	Apoptosis (% TUNEL positive cells)
Western diet (WD)	29.2 ± 7.3%	8.0 ± 2.7
WD + ginseng	15.3 ± 4.6%*	11.9 ± 4.0†

*p < 0.001, compared to Western diet alone; †p < 0.05, compared to WD alone.

### Effects of ginseng on EGFR signals and regulators of apoptosis

**Figure 3:**
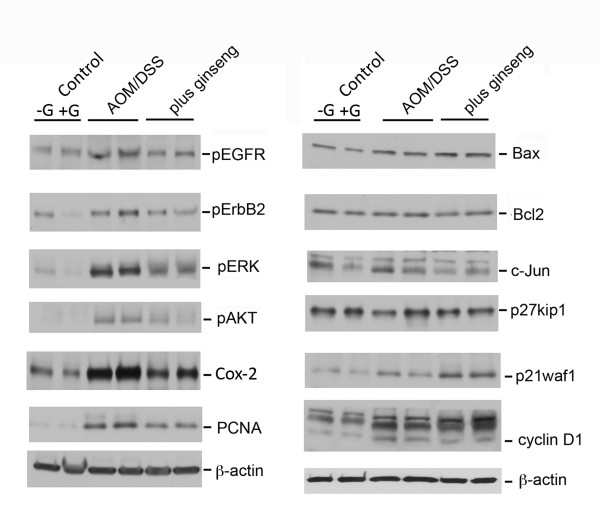
**Ginseng inhibits EGFR signals and Cox-2 up-regulation and increases pro-apoptotic Bax in AOM/DSS tumors**. Control mucosa (-G, Western diet alone, +G, Western diet + ginseng) and colonic tumors were homogenized in Laemmli buffer and indicated proteins detected by Western blotting. Shown are representative control samples and tumors from each group. Quantitative densitometry is provided in Table 2.

To begin to dissect molecular signals potentially contributing to ginseng-induced changes in proliferation and cell death, we examined EGFR signals and apoptosis regulators. As shown in Figure 3, colonic tumors induced by AOM/DSS in mice on Western diet showed up-regulations of phospho-active-EGFR (pEGFR), pErbB2, pERK, and pAKT (Figure 3). Dietary ginseng significantly reduced these activations in tumors. Ginseng also increased cell cycle and apoptosis regulating p21Waf1 in tumors that is predicted to inhibit proliferation and increase apoptosis [45,46]. With respect to other apoptotic regulators, ginseng decreased anti-apoptotic Cox-2 and increased pro-apoptotic Bax, consistent with ginseng-induced increased apoptosis. Quantitative densitometry values are summarized in Table 2.

**Table 2:** EGFR signals and apoptosis mediators

Protein	AOM/DSS	+ginseng
pEGFR	2.2 ± 0.3*	1.1 ± 0.2†
pErbB2	2.8 ± 0.4*	1.6 ± 0.1†
pERK	8.3 ± 0.1*	5.8 ± 0.3*,†
pAKT	7.2 ± 0.3*	4.4 ± 0.8*,†
Cox-2	3.1 ± 0.3*	1.9 ± 0.2*,†
PCNA	10.7 ± 1.2*	6.3 ± 0.1*,†
Bax	1.6 ± 0.3	2.0 ± 0.1*,†
Bcl2	1.1 ± 0.1	1.1 ± 0.2
c-Jun	1.2 ± 0.2	0.6 ± 0.2†
p27Kip1	1.0 ± 0.5	0.9 ± 0.1
p21Waf1	1.8 ± 0.2	3.8 ± 0.1*,†
CCND1	2.9 ± 0.6*	2.7 ± 0.4*

*p < 0.05, compared to vehicle treated on WD; †p < 0.05 compared to AOM/DSS on WD; n = 4 tumors in each group.

### Absorption and biological effects of ginseng metabolite compound K

**Figure 4:**
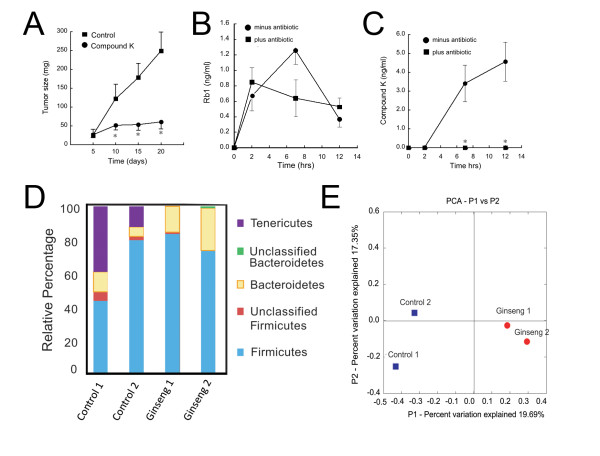
**Dietary ginseng alters the composition of the bacterial flora while intraperitoneal compound K inhibits colon cancer xenograft growth**. **A**. Compound K inhibits colon cancer growth in tumor xenograft. HCT116 cells (5*10^6 cells) were implanted into flanks of *nu/nu *mice and allowed to grow for 5 days. Mice were then treated with compound K (30 mg/kg-body wt or DMSO (compound K vehicle). At indicated times tumor size was estimated by linear dimensions [volume = 1/2 × length × (width)^2^] and expressed in mg (*p < 0.05 compared to control, n = 5 mice in each group). **B**. Rb1 absorption. Mice were un-treated or treated with metronidazole in the drinking water. After 5 days animals were gavaged with 500 mg ginseng extract/kg body wt. At indicated times after ginseng gavage, plasma levels of ginsenoside Rb1 were measured by UPLC-mass-spectrometry/TOF. **C**. Compound K absorption. Mice were treated as described in 4B and Compound K (C-K) measured by UPLC-mass-spectrometry/TOF. Data were expressed as means ± SEM depicted by vertical bars (p < 0.05 compared to mice not receiving antibiotics). Note that metronidazole suppressed serum compound K but not Rb1 levels. **D**. Effects of ginseng on bacterial phyla. Mice were fed Western diet or Western diet containing 250-ppm ginseng. After 2 wks feces were collected and bacterial DNA extracted. Using 16S rRNA gene libraries, bacteria were classified by phyla as described in "Materials and Methods". **E**. Principal coordinate analysis (PCA) of bacterial distributions. Note that ginseng decreased the abundance of Tenericutes phylum and appeared to widely separate species as assessed by PCA.

Several metabolites of ginseng require colonic microbiota for biosynthesis [47]. In recent preliminary studies we found that whereas the ginsenoside Rb1 had limited anti-proliferative and pro-apoptotic activity, 20-O-β-(D-glucopyranosyl)-20(S)-protopanaxadiol or compound K, a microbial metabolite of Rb1 potently suppressed colon cancer cell proliferation *in vitro *(C. Wang *et al.*, manuscript submitted). To directly assess the anti-tumor effects of compound K we examined the ability of this bacterial metabolite of ginseng to inhibit tumor xenograft growth. The structure of compound K is shown in Additional File 1. As shown in Figure 4A, compound K potently suppressed growth of HCT116 cells in immunodeficient nude (*nu/nu*) mice when administered by intraperitoneal route.

To address a colonic bacterial requirement for compound K synthesis we pretreated mice with metronidazole a broad-spectrum antibiotic or vehicle (control). Following 5 days treatment we measured ginsenoside Rb1 and compound K in the sera by mass spectrometry. As shown in Figure 4B, Rb1 absorption was not influenced by antibiotic treatment. In contrast, compound K was undetectable in antibiotic-treated mice, but readily detected in mouse sera from vehicle-treated mice (Figure 4C). These results are consistent with a requirement for metronidazole-sensitive colonic bacteria for compound K synthesis.

### Ginseng alters colonic microbiome diversity

While bacteria are required for metabolism of some ginsenosides, it is also possible that ginseng might alter the microbial flora since it is a substrate for some bacteria. To assess the effects that ginseng might have on the microflora, we compared colonic bacterial populations in mice on 2 wks of Western diet alone vs. those on Western diet plus 250 ppm ginseng extract. As shown in Figure 4D, ginseng reduced the number of *Tenericutes*, while increasing the *Bacteroidetes *population. Principal coordinate analysis suggested that the microbial populations in mice receiving ginseng were markedly distinct from those on Western diet alone (Figure 4E). These changes support our hypothesis that ginseng consumption might alter the colonic microbiome. Whether these changes influence microbial metabolic activity towards ginseng, including compound K biosynthesis, will require further study.

## Discussion

We have shown for the first time that an extract of ginseng can inhibit colonic tumor development under conditions of a Western diet in a model of inflammation-associated colon cancer. These studies extend recent findings that ginseng can inhibit colonic tumorigenesis under standard dietary fat conditions [19]. We also observed that ginseng delayed the onset and appeared to lessen the severity of inflammation in this model in agreement with a prior study [48]. Since inflammation plays a key role in this model, the anti-cancer effects of ginseng likely derive in part from its ability to suppress inflammation, as also suggested by ginseng suppression of Cox-2 a molecular marker of inflammation [49]. Cox-2 inhibition has been shown to directly suppress tumorigenesis in this model [50]. Ginseng appeared to inhibit tumor initiation, as tumor multiplicity and the number of adenomas were significantly reduced in the ginseng treated group. There was also a trend towards fewer cancers in the ginseng group, suggesting that this agent might inhibit tumor progression, but this did not reach statistical significance. In a study under standard dietary fat conditions ginseng inhibited tumor progression [19].

Ginseng supplementation reduced proliferation and increased apoptosis in tumors. Compared to tumors from the unsupplemented group, decreased PCNA expression in the ginseng group was consistent with reduced proliferation. Prior studies by our group and others have shown that ginseng or its metabolites induce anti-proliferative and pro-apoptotic effects in human cancer cells [12,15,31,51,52]. More recently, we demonstrated that compound K, a microbial metabolite of ginsenoside Rb1, induced G1 cell cycle slowing and accelerated apoptosis of colon cancer cells (C. Wang *et al.*, manuscript submitted). Compound K has also been shown to inhibit growth and increase apoptosis in a model of liver cancer metastasis [27].

To begin to elucidate cellular signaling pathways that mediate these anti-proliferative and pro-apoptotic effects we examined expression levels of several proto-oncogenes and tumor suppressors. We found in tumors from the unsupplemented group that EGFR signals, including pEGFR, pErbB2, pERK and pAKT were significantly increased. Ginseng significantly reduced increases in EGF receptor activation and inhibited these down-stream effectors that are known to drive mitogenic and pro-survival signals in colon cancer [53,54]. Ginseng also increased p21Waf1, a cyclin-cdk inhibitor predicted to retard G1 - > S cell cycle progression. While we did not measure changes in p53, a major regulator of p21Waf1, this cyclin-cdk-dependent inhibitor can also regulate p53-independent pathways [46].

Among the regulators of apoptosis, we demonstrated that ginseng significantly reduced Cox-2 and increased pro-apoptotic Bax in tumors. Cox-2 is an EGFR effector in this model that suppresses apoptosis in colon cancer cells [4,55]. This pro-inflammatory molecule plays a critical role in both sporadic and inflammation-associated colonic tumorigenesis [56,57]. With respect to Bax, down-regulation of this protein has been suggested to predict colon cancer prognosis in early stage disease [58]. Changes in these regulators were consistent with increased apoptosis in tumors from ginseng-supplemented mice. Since we showed that EGFR signals were required for Western diet to promote colonic tumorigenesis [4,5], we speculate that inhibition of this cascade plays a critical role in the chemopreventive effects of ginseng by limiting proliferation and increasing apoptosis in this model. As this commonly used and safe natural herb appears to inhibit diet-promoted colon cancer, ginseng might offer a novel chemopreventive strategy for colon cancer, particularly in Asia where there is widespread use of ginseng and increasing adoption of Westernized diets.

In recent preliminary *in vitro *studies we showed that the anti-proliferative and pro-apoptotic effects of the ginsenoside Rb1 were likely mediated by compound K, a major microbial metabolite of Rb1. In agreement with these findings, others have shown that compound K induced apoptosis in colon cancer cells *in vitro *and inhibited hepatocellular tumor xenograft growth *in vivo *[27,28]. Compound K is derived from Panax ginsenoside Rb1 by the microbial enzyme geniposide-hydrolysing beta-D-glucosidase [47]. Antibiotic treatment suppressed the appearance of compound K in the serum. Rb1 and compound K appear to block IRAK-1 and NFκB activation and thereby reduce pro-inflammatory cytokines, IL-1β, TNF-α and IL-6 and cytokine effectors iNOS and Cox-2 in 2,4,6-trinitrobenzene sulfonic acid (TNBS)-treated mice, another model of colitis [59]. To directly assess the anti-tumor effects of compound K in a colon cancer model we examined the effect of this compound on HCT116 tumor xenograft growth. Compound K significantly inhibited colon cancer growth. We speculate, therefore, that this microbial metabolite contributes to the chemopreventive efficacy of ginseng in the AOM/DSS model. We plan to directly test this assertion in future studies using germ-free animals.

It will also be of interest to determine how host-genetic and environmental factors interact with the colonic microbiome. In contrast to a bacterial requirement for compound K generation, bacteria do not appear to be necessary to activate AOM as colonocytes from germ free rats generated AOM from precursor DMH at least as fast as colonocytes from conventionally colonized rats [60]. Furthermore, AOM can induce tumors in germ free rodents [61]. In addition to an effect of the microbes on ginseng metabolism, ginseng in the diet might reshape the microbiome by serving as a metabolic substrate or an environmental modifier to enrich microbial species capable of using or responding to this agent. This could lead to increases in the capacity to metabolize ginseng to bioactive molecules such as compound K. Thus, sustained use of ginseng by individuals might promote clinical efficacy by increasing metabolic conversion of relatively inert ginseng to bioactive components. Such investigations might suggest more effective dietary and probiotic approaches to enhance ginseng-based colon cancer chemoprevention.

## Conclusions

Ginseng inhibits AOM/DSS induced colonic tumorigenesis promoted by a Western diet. Inhibition of EGFR signals and alterations in G1 cell cycle and apoptosis regulators appear to contribute to these chemopreventive effects. Studies in tumor xenografts suggest that compound K, a microbial metabolite of ginseng, may mediate some of these effects of ginseng. Further study of this promising natural agent for chemoprevention of colon cancer is warranted.

The abbreviations used are: AOM, azoxymethane; compound K, 20-O-β-(D-glucopyranosyl)-20(S)-protopanaxadiol; Cox-2, cyclooxygenase-2; DAI, disease activity index; DSS, dextran sulfate sodium; EGFR, epidermal growth factor receptor; PCA, principal coordinate analysis; TUNEL, terminal transferase dUTP nick end labeling; UPLC-MS/TOF, ultra performance liquid chromatograph mass spectrometry/time of flight; WD, Western diet.

## Competing interests

The authors declare that they have no competing interests.

## Authors' contributions

UD carried out the AOM/DSS experiments and tumor xenograft studies and tissue Western blotting and immunostaining and assisted with mouse colonoscopy. RM prepared HCT116 cells for tumor xenograft studies. YW extracted fecal microbial DNA, prepared clone libraries and sequenced 16S rDNA. He also carried out sequence alignment and phylogenetic characterization using principal coordinate analysis. MWM helped conceive the study, and participated in its design and coordination and helped draft the manuscript. CSW extracted ginseng and analyzed dietary ginsenoside content and measured ginsenosides in blood. VJK carried out mouse colonoscopies and endoscopic assessment of inflammation and tumors. AK assisted VJK with colonoscopies and assisted UD with tissue sectioning and staining. JH provided all histological assessments of inflammation and neoplasia. GD assisted with ginseng extraction and purification and study design and interpretation. KEK helped conceive the study and helped draft the manuscript. CSY helped conceive the study, participated in its design and guided ginsenoside analyses and helped draft the manuscript. EBC helped conceive the study, participated in its design and coordination, guided analysis of the microbiome and helped draft the manuscript. MB helped conceive the study, participated in its design and coordination, guided the in vivo AOM/DSS and tumor xenograft studies and helped draft the manuscript. All authors read and approved the final manuscript.

## Pre-publication history

The pre-publication history for this paper can be accessed here:

http://www.biomedcentral.com/1472-6882/11/111/prepub

## Supplementary Material

Additional file 1 **Compound K**. A TIF image of the structure of compound K. Click here for file
