# Supplementary figures and images for: American ginseng suppresses Western diet-promoted tumorigenesis in model of inflammation-associated colon cancer: role of EGFR
Source: BMC Complement Altern Med. 2011 Nov 9;11:111. doi: 10.1186/1472-6882-11-111 (PMC3227598; doi:10.1186/1472-6882-11-111)

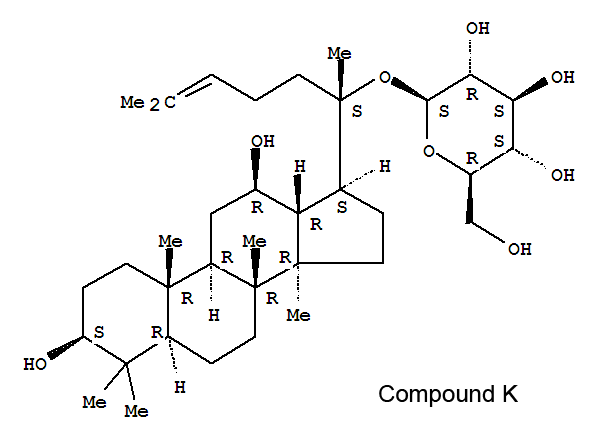

Supplement: Additional file 1 — Compound K. A TIF image of the structure of compound K. [file 1472-6882-11-111-S1.TIFF]
